# Formulation and Characterization of Curcumin Niosomes: Antioxidant and Cytotoxicity Studies

**DOI:** 10.3390/ph16101406

**Published:** 2023-10-03

**Authors:** Shazia Akram Ghumman, Amna Ijaz, Sobia Noreen, Afeefa Aslam, Rizwana Kausar, Ali Irfan, Sumera Latif, Gamal A. Shazly, Pervaiz Akhtar Shah, Maria Rana, Asma Aslam, Momina Altaf, Katarzyna Kotwica-Mojzych, Yousef A. Bin Jardan

**Affiliations:** 1 College of Pharmacy, University of Sargodha, Sargodha 40100, Pakistan; 2 Institute of Chemistry, University of Sargodha, Sargodha 40100, Pakistan; 3 Abbottabad Campus, COMSATS University Islamabad, Abbottabad 22060, Pakistan; 4 ILM College of Pharmaceutical Sciences, Sargodha 40100, Pakistan; 5 Department of Chemistry, Government College University Faisalabad, Faisalabad 38000, Pakistan; raialiirfan@gmail.com; 6 Institute of Pharmacy, Faculty of Pharmaceutical and Allied Health Sciences, Lahore College for Women University, Lahore 54000, Pakistan; 7 Department of Pharmaceutics, College of Pharmacy, King Saud University, Riyadh 11451, Saudi Arabia; 8 University College of Pharmacy, University of the Punjab, Lahore 54590, Pakistan; 9 Riphah Institute of Pharmaceutical Sciences, Riphah International University, Lahore 54000, Pakistan; 10 Laboratory of Experimental Cytology, Medical University of Lublin, Radziwiłłowska 11, 20-080 Lublin, Poland

**Keywords:** curcumin, niosomes, Box–Behnken, antioxidant, cytotoxicity, ovarian cancer

## Abstract

Curcumin’s applications in the treatment of conditions including osteoarthritis, dementia, malignancies of the pancreas, and malignancies of the intestines have drawn increasing attention. It has several wonderful qualities, including being an anti-inflammatory agent, an anti-mutagenic agent, and an antioxidant, and has substantially reduced inherent cytotoxicity outcomes. Although curcumin possesses multiple known curative properties, due to its limited bioavailability, it is necessary to develop efficient strategies to overcome these hurdles. To establish an effective administration method, various niosomal formulations were optimized using the Box–Behnken design and assessed in the current investigation. To examine the curcumin niosomes, zeta sizer, zeta potential, entrapment efficiency, SEM, antioxidant potential, cytotoxicity, and release studies were performed. The optimized curcumin niosomes exhibited an average particle size of 169.4 nm, a low PDI of 0.189, and high entrapment efficiency of 85.4%. The release profile showed 79.39% curcumin after 24 h and had significantly higher antioxidant potential as compared with that of free curcumin. The cytotoxicity results of curcumin niosomes presented increased mortality in human ovarian cancer A2780.

## 1. Introduction

Curcumin or diferuloylmethane is a natural chemical compound; its source is Curcuma longa. Curcumin has multiple medicinal benefits, including anticancer, antioxidant [1], and anti-inflammatory properties [2]. Several investigations showed evidence of curcumin having a protective effect in cerebral ischemia [3]. Furthermore, curcumin modulates several molecular pathways within cancer cells, including interfering in the activation of protein kinase D1, and may help to prevent the development of tumor growth and the onset of mortality [4,5]. However, curcumin exhibits poor bioavailability, low aqueous solubility, and fast in vivo deterioration owing to its chemical structure, which limits its clinical use [6,7]. It is safe even at high doses of up to 12 g/day. After oral ingestion, no curcumin is detected in urine; however, it shows a large amount of excretion in the feces due to its metabolic clearance through reduction and conjugation [8]. Hence, it is difficult to reach appropriate curcumin blood levels that might increase its significant beneficial therapeutic advantages. To increase medication solubility and uptake as well as reduce turmeric degradation and excretion, a novel drug delivery system is needed [9].

A controlled drug delivery system relies on a specific method to transfer a certain amount of a drug for a prolonged time; in this way, the required plasma and tissue drug concentration is maintained inside the body while protecting healthy tissues from damage caused by the drug [10]. Niosomes, immunoglobulins, microspheres, serum proteins, liposomes, and erythrocytes are several carriers employed for novel drug delivery systems [11]. Because of their unique advantages, niosomes gained a lot of attention during the last 30 years as a potential drug delivery system [12,13]. Niosomes are capable of encasing hydrophilic drugs in aqueous divisions or lipophilic drugs by dividing these molecules into bilayer areas due to their versatility in framework, shape, and measurement [14]. While contrasted with other vesicle-based drug delivery systems that need complicated linking techniques to capture substances, making it challenging to integrate all medications, niosomes have benefits over other vacuoles-based systems for delivering drugs, and their simplicity of production is appropriate regarding the simple trapping of an active pharmaceutical ingredient. When it comes to durability and in general distribution and duration of action, niosomes perform better than liposomes in additional aspects [15].

The present study aimed to prepare a niosomal delivery system to improve the solubility and bioavailability of curcumin by its entrapment into a vesicle system. We prepared and characterized curcumin niosomes through particle size, polydispersity index, zeta potential, and morphology of loaded curcumin niosomes by scanning electron microscopy. We further determined the physical state of curcumin in niosomes by thermal analysis and interaction between curcumin and different components of niosomes by Fourier transform infrared spectroscopy (FTIR). We also assessed the effect of different non-ionic surfactants while keeping the proportion of cholesterol constant.

## 2. Results and Discussion

### 2.1. Formation and Optimization of Curcumin Niosomes (Curcusomes) by Box–Behnken Design (BBD)

**Figure 1:**
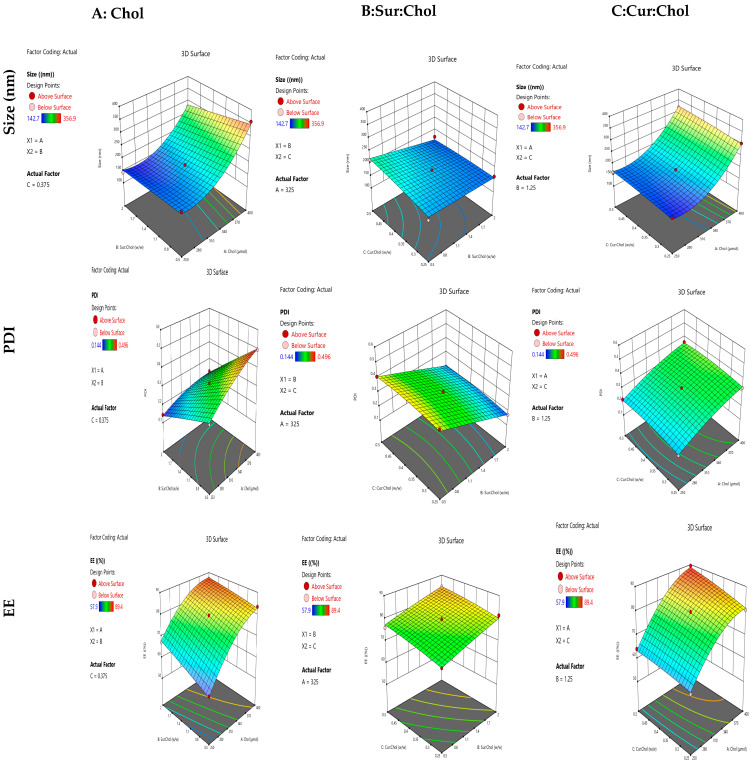
Three-dimensional response plots display the influence of independent variables on (**A**) particle size, (**B**) PDI, and (**C**) entrapment efficiency of curcusomes (*n* = 3).

All the trial runs generated by Box–Behnken was performed and characterized in terms of vesicle size, PDI, and EE. The responses from fifteen trials are shown in Table 1. The effect of independent variables on curcusome size, polydispersity index (PDI), and entrapment efficiency (EE) is also presented as three-dimensional response surface graphs in Figure 1). From the experiments, it was observed that the amount of cholesterol, the surfactant–cholesterol ratio, and the curcumin–cholesterol ratio had a marked influence on size, PDI, and EE. 

Among the tested formulations, F2, composed of 325 μmol of cholesterol, with a ratio of surfactant to cholesterol of 2:1, and 0.5% w/w was curcumin considered optimal based on the smaller size (169.4 nm), low PDI (0.189), and high EE (85.4%).

#### 2.1.1. Influence of Independent Variables on Size

The sizes of the curcusomes were between 142.7 and 356.9 nm in diameter. An increase in cholesterol concentration demonstrated an increase in size. The mean diameter of the two separate layers that constitute niosome formation likewise grows when cholesterol levels rise [16,17].

The regression analysis of all variable is given in Table 2. Surfactants had significant negative effects on PS as depicted by the *p*-value, which was observed (*p* < 0.05). A steric shield was created across the outermost layer due to the lipid nanoparticles stabilized by a large quantity of surfactants. As an outcome, the particle size was small.

The polynomial equation obtained with Design-Expert software intended for size was as follows:

Size = 764.669 − 5.32839 × A + 86.0667 × B + 331.367 × C − 0.209333 × AB + 0.176 × AC − 122.933 × BC + 0.0100844 × A^2^ + 0.533333B^2^ − 203.2 × C^2^

#### 2.1.2. Influence of Independent Variables on PDI

PDI is a suitable indicator of the stability of a system. Nanocarriers with a low PDI have superior stability and a homogenous size distribution. The PDI values obtained from all experiment results ranged from 0.144 to 0.496. RSM graphs shown in Figure 1B indicate the effect of the independent variables on the PDI. As the Chol concentration got higher, the lipid phase’s raised viscosity led to poor homogeneity. That describes how particle clustering increased the size of the particles and the PDI.

Surfactants (Tween 80 and Span 80) had a negative effect on the PDI (*p*-value < 0.05). As revealed in Figure 1B, a reduction in PDI was perceived when Sur ratio increased. The surface tension between the two phases decreases with a rise in Sur quantity, prohibiting the agglomeration of particles [18]. The PDI readings determined for the prepared niosomes with different Cur to Chol ratios varied by a small amount. The polynomial equation obtained from Design-Expert software for PDI was as follows:

PDI = −0.578486 + 0.00414444 × A + 0.0275556 × B + 0.644 × C − 0.000564444 × AB + 8 × 10^−5^ × AC + 0.056 × BC −3.84444 × 10^−6^ × A^2^ − 0.00377778 × B^2^ + −0.904 × C^2^

#### 2.1.3. Influence of Independent Variables on EE

All three independent variables had major effects on (*p*-value < 0.05) as described in Table 2. The EE of all formulations varied from 57.9 to 89.4. It can observed in Figure 1C that all independent variables had a positive effect on EE.

EE was influenced by cholesterol content, and a direct link between cholesterol content and EE was observed. The presence of cholesterol increased the entrapment efficiency by reducing liquid–gel transition, allowing vesicles to incorporate more drugs. Furthermore, a high quantity of Chol decreased the possibility of drug leakage into the aqueous phase from the hydrophobic carrier, leading to a high percentage of EE.

Furthermore, as the Cur to Chol proportion increased, EE also increased, which may have been because there was accessibility to a larger space for the drug to stay in the core. Hence, it can concluded that by increasing the amount of Chol, Sur, and Cur, would increase.

The polynomial equation obtained from Design-Expert software for EE was as follows:

EE = −77.9574 + 0.716574 × A + 15.7093 × B − 1.73333 × C − 0.0266667 × AB + 0.146667 × AC − 1.86667 × BC − 0.000903704 × A^2^ + −0.503704 × B^2^ − 32.5333 × C^2^

Similar responses were reported for dexamethasone encapsulated in niosomes where an increase in vesicle size and percentage of EE of dexamethasone in niosomes was observed with an increase in cholesterol content and decreased surfactant to cholesterol ratio [19]. The model’s F-values for the dependent factors size, PDI, and EE were 50.25, 132.42, and 96.25, respectively. These values indicate the significance of the experimental model. Based on the highest R^2^ values, the quadratic effect was the suggested model for all dependent variables in comparison to cubic 2FI and the linear effect. This indicates that the quadratic model was suitable for the experimental design. The *p*-value for the lack-of-fit test was found to be >0.05 for all variables that described the suitability of the model. Moreover, the coefficient of variation (% CV) values were 5.72, 3.75, and 1.61 for size, PDI, and EE, respectively. A % CV value below 10 indicates that a model is reliable and reproducible. The composition of the optimized curcusomal formulation suggested by Design-Expert^®^ 13 software is described in Table 3. The observed responses were nearly identical to the values predicted by the design, showing that the optimization and prediction processes were valid.

### 2.2. Characterization of Optimized Formulation

#### 2.2.1. Size Distribution, Surface Charge, and Morphology

**Figure 2:**
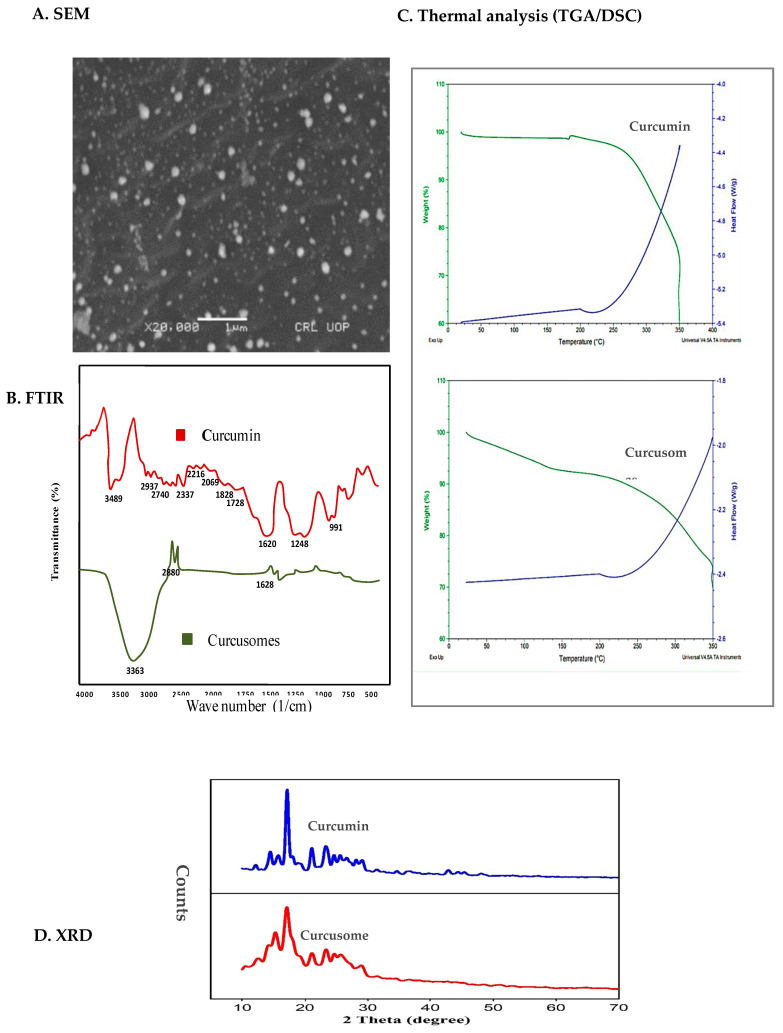
Characterization of curcusomes’ optimized formulation: (**A**) SEM morphological image; (**B**) FTIR spectra; (**C**) TGA-DSC spectra; (**D**) XRD diffractograms.

The size distribution of the optimized curcusomal formulation is shown in Figure S1A (Supplementary Materials). A single narrow peak below 200 nm and a low PDI value indicated homogeneity in the size distribution. Surface charge is measured in terms of zeta potential. Dispersions with values greater than 30 mV are considered as highly stable [20]. The zeta potential of the optimized formulation was found to be −50.2 mV (Figure S1B). The negative charge might be due to better adsorption of hydroxyl ions on the vesicle’s surface. Figure 2A shows the surface morphology of curcusomes. The particles were circular with well-defined boundaries.

#### 2.2.2. FTIR

The chemical compatibility of the components of curcusomes was determined by FTIR. Figure 2B shows the FTIR spectrum of pure Cur and Cur-loaded formulation. The peaks observed for pure Cur at 3489 cm^−1^, 1620 cm^−1^, 1248 cm^−1^, and 991 cm^−1^ corresponded to stretching of the O-H group in the benzene ring, C=C aromatic ring stretching, and C-O-C symmetric stretching vibration of the methoxy group. The spectrum of curcusomes exhibited peaks of the functional groups of cholesterol, Tween 80, Span 80, and Cur. The most characteristic absorption peaks from cholesterol and surfactants corresponded to alcohols and ester functional groups [21]. The peak due to Cur also appeared in the spectrum of curcusomes at 1628 cm^−1^ [22]. The results specified that Cur was encapsulated well in the curcusomes, showing no interaction.

#### 2.2.3. DSC/TGA

Thermal analysis of pure Cur and curcusomes was done by obtaining simultaneous DSC/TGA curves. The thermograms of both samples were similar, as shown in Figure 2C. The DSC curve of Cur presented a sharp melting endotherm around 200 °C, representing its crystalline nature. The TGA curve of pure curcumin showed a small peak at 177.4 °C due to the melting peak of Cur. The peak did not appear in the TGA curve of curcusomes, which showed that Cur was entrapped in the lipid matrix in amorphous form. This fact was also confirmed by a marked reduction in enthalpy for curcusomes compared to that of pure curcusomes. All these findings established the thermal stability of the curcusomes.

#### 2.2.4. X-ray Diffraction (XRD)

The XRD spectrum of pure Cur and curcusomes is displayed in Figure 2D. The spectrum of Cur exhibited sharp characteristic peaks at scattering angles of 16.2, 17.6, and 21.2, indicating its crystalline nature. However, a marked drop in the strength of these peaks was detected in the spectrum of the curcusome, which indicated a loss of its crystalline nature. It is reported in the literature that drugs disperse more easily when in amorphous form, thus having improved bioavailability. The amorphous nature of a drug is also linked to its stability, which is enhanced by this state of curcumin [23].

#### 2.2.5. In Vitro Drug Release and Kinetics

**Figure 3:**
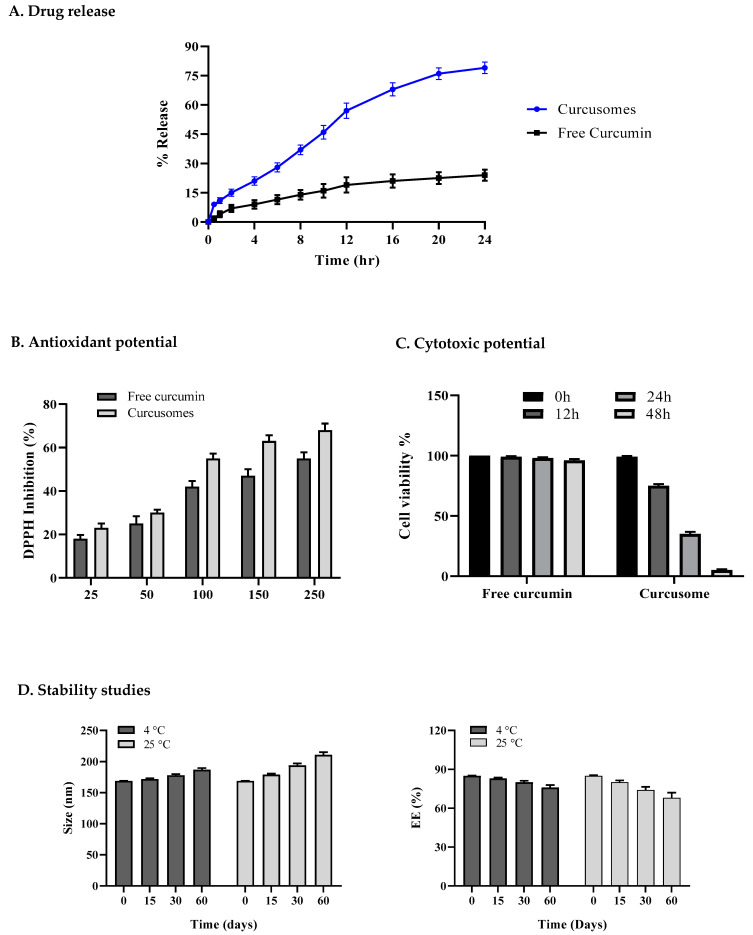
(**A**) Comparative drug release profiles of free curcumin and curcusomes at pH 7.4, *n* = 3; (**B**) antioxidant potential; (**C**) cytotoxic potential; and (**D**) stability of optimized curcusomal formulation at different temperatures (4 and 25 °C).

The release of curcumin from niosomes displayed a pattern of persistent sustained release as shown in Figure 3A. The drug release percentage from curcusomes was 79.39% after 24 h, signifying that niosomes delivered a tremendous release from the polymers. To study the release kinetics, numerous mathematical models were used, i.e., first-order, zero-order, Higuchi, and Korsmeyer–Peppas models. The results showed that the curcusomes (pH 7.4) followed the Korsmeyer–Peppas model with an R^2^ value of 0.9906 and showed a diffusion-controlled mechanism [24]. The delayed degradation of the lipid bilayer, which suggested exceptionally stable behavior of the nanocarrier, may be the reason for the drug’s persistent discharge out of the niosomal framework [25].

#### 2.2.6. Antioxidant Potential

In this investigation, the capacity of every sample to scavenge free radicals was assessed via observation of the alteration in absorption brought about by the decrease in DPPH. The percent inhibition of different amounts of curcusomal formulation and free curcumin ethanolic solution against DPPH is shown in Figure 3B. According to the results, curcusomes had significantly higher antioxidant activity than free curcumin did. This enhanced effect could be attributed to the higher solubility of curcumin in niosomal preparations. Curcumin exhibits an antioxidant effect, especially due to its phenolic hydroxyl groups.

#### 2.2.7. Cytotoxic Potential

The cytotoxicity of curcusomes after 0, 12, 24, and 48 h of incubation is displayed in Figure 3C. Curcusomes showed substantially increased cell death compared to free curcumin. Within 48 h of being incubated via curcusomes, over 90% of the cells died. Curcumin niosomes showed considerable mortality in contrast to that with free curcumin, and that was probably because of the better solubility of curcumin in water and increased cellular absorption [11].

#### 2.2.8. Stability Studies

The stability of the stored curcusomes was evaluated every 15 days for over 60 days at 4 °C (refrigerator) and 25 °C (room temperature). There seemed to be no noticeable changes in curcusomes’ size and EE in the stored and freshly prepared samples. This revealed that curcusomes exhibited excellent physical stability when kept at 4 °C, which was attributable to the fact that the cold temperature prevented the disintegration of the niosomes. The high stability could be attributed to increased zeta potential and excellent electrostatic repulsion strength among vesicles [26].

### 2.3. Drug–Excipients Compatibility Study

FTIR studies were carried out to check drug–excipient compatibility. It was evident from the spectra that all three polymers did not interact with the drug [27,28,29].

## 3. Experimental

### 3.1. Materials

Curcumin, cholesterol, Span 80, and Tween 80 were procured from Sigma-Aldrich, Darmstadt, Germany. Analytical grade ethanol, and chloroform were acquired from Fisher Chemical, Waltham, MA, USA. A dialysis membrane (12 kD) was obtained from Spectrum Chemicals, NJ, USA.

### 3.2. Methods

#### 3.2.1. Preparation of Curcusomes

Curcusomes were formulated with thin-film hydration followed by sonication, as stated formerly and using minor adjustments [29,30]. Briefly, different amounts of surfactant (Span80–Tween80, 70:30 *v*/*v*) and cholesterol were completely dissolved in chloroform (8 mL) and ethanol (2 mL) in a dry round-bottom flask, followed by the addition of curcumin in this mixture. The solvent evaporated with continuous rotations in a rotary evaporator (Buchi rotary evaporator) at 40 °C and 150 rpm to produce a fine lipid layer. Afterwards, the dried thin films were hydrated in ultra-pure water (10 mL, pH 7.4). Subsequently, a sonicator bath (Labtech International, Winchester, UK) was used to shake the samples for 30 min to produce the curcusomes with a uniform size distribution. To separate unloaded curcumin, the formulation was dialyzed using a dialysis bag (12 kDa MWCO). The prepared curcusomes remained in a refrigerator (4 °C) until further investigation [27,31].

#### 3.2.2. Optimization of Curcusomal Preparations with Box–Behnken Design

The Box–Behnken experimental design was used with Design-Expert 13.0.10 software (Stat-Ease Inc., Minneapolis, MI, USA) for the investigation of the independent factors’ effects (amount of cholesterol—X_1_, surfactant to cholesterol ratio—X_2_, curcumin to cholesterol ratio—X_3_) on the physicochemical properties of curcusomes. The 15 trials with three duplicated center points were proposed by RSM. Based on initial investigations and studies, the highest and lowest values of the parameters were chosen [18]. The minimal, middle, and maximal values for each of these independent variables are displayed in Table 4. The influence of these variables (responses) was examined on droplet size (Y_1_), polydispersity index (Y_2_), and entrapment efficiency (Y_3_). Furthermore, 3D responsive surface plots are displayed. The formulation resulting in curcusomes with a small size, minimal PDI, and significant EE was deemed the most efficient and was employed in additional tests.

#### 3.2.3. Characterization of Optimal Formulation

##### Size Distribution Analysis

Malvern Zeta Sizer (Malvern Panalytical Ltd, Malvern, UK) used the method known as dynamic light scattering (DLS) technology to measure the median droplet dimensions and the relative diameters of curcusomes. To prevent numerous dispersion phenomena, curcusome compositions were properly blended (1:10) with the use of deionized water. The sample was placed in a 10 × 10 × 45 mm polystyrene cell and, using an ambient temperature of 22 °C with a scattering angle of 90°, every test was performed in duplicate (*n* = 5) [32].

##### Entrapment Efficiency

Entrapment efficiency (EE) was measured by centrifuging (SIGMA 3-30 KS, Hessen, Germany) 1 mL of each suspension at 12,000× *g* for 15 min, followed by filtration of the supernatant. The supernatant was investigated using a spectrophotometer at a 425 nm wavelength [33].

$$\%EE=\frac{\left[A-B\right]}{A}\times 100$$

where *A* is the initial amount of curcumin taken for the curcusomal formulation and *B* is the amount of free curcumin present in the supernatant [34].

##### X-ray Diffraction (XRD)

For XRD analysis a Bruker D8 Discover (Billerica, MA, USA) diffractometer fortified with a proportionate stand, using Cu-Ka emission (l = 1.5405 Å, nickel filter), was used. The experiment was performed using a voltage of 30 kV and an associated current of 40 mA. Data were collected using samples on a silicon wafer slide in the range 2θ = 10° to 100°, scanning at 1.5° min^−1^ through 0.38 s/step and a width of 6.0 mm [15].

##### Chemical Compatibility of Formulation Components (FTIR)

Compatibility between the drug and other components of the vesicular formulation was determined by this technique: FTIR was employed to determine the spectra of curcumin, cholesterol, the physical mixture, and the final formulation of niosomes within the range of 5000–400/cm^−1^ wave number [35]. A solid sample was examined by the KBr method, and percent transmission was recorded.

##### Thermal Analysis (DSC/TGA)

A freeze-dried sample of the optimal formulation was analyzed by the SDT-TA instrument. In an aluminum pan, 2–5 mg of each specimen was tightly packed before being subjected to an ambient nitrogen analysis using a temperature between 30 and 300 °C and an analysis rate of 10 °C/min. An empty aluminum pan was employed as the baseline value while taking the measurements [15].

##### Morphology

A scanning electron microscope (Carl Zeiss, EVO43, SEM, Oberkochen Germany) was employed to examine the morphology of the optimized formulation. One hundred microliters of the best formulation was diluted in 1 mL of deionized water and afterwards, 10 µL of the specimen was placed on a slide of glass and then left to dry completely. Images were captured after a specimen was sputtered with 100 nm gold fragments for three minutes in an environment of argon in a highly vacuumed evaporation device [36].

##### Surface Charge Measurement

Zeta potential was calculated to assess the stability and surface charge of the optimized formulation with a Zetasizer Nano ZS at 22 °C (Malvern Instrument Ltd. Malvern, UK). A tiny vessel was used for the testing of 1 mL of the specimen [37].

##### Storage Stability

To determine stability, the best preparation remained in two different environments (25 ± 1 °C and 4 ± 1 °C) for 60 days, and the alterations in droplet size and EE in the preparations were estimated at precise period intermissions (15 days) [30].

##### In Vitro Drug Release and Kinetics

For the release study, 5 mL of the optimized formulation was sealed in a semi-permeable cellulose membrane dipped in 50 mL of phosphate buffer of pH 7.4 with constant stirring of 50 rpm at 37 °C for 24 h. With precise time intervals, 1 mL of the sample was taken and substituted by a similar volume of fresh medium. The sample was determined with UV–visible spectroscopy at 425 nm wavelength. The experiment was performed three times, and the mean of both values was taken into consideration [38]. The data obtained from in vitro drug release studies was fitted into different models, namely zero-order, first-order, Higuchi, and Korsmeyer–Peppas models in order to understand the drug release mechanism [39].

##### Antioxidant Potential

A DPPH antioxidant experiment was carried out for a curcumin ethanol-based solution, curcumin niosomes, and blank niosomes. Concisely, 1 mL of DPPH ethanol-based solution (0.2 mM) and 1 mL of curcumin niosomal dispersion were blended (niosomal dispersion samples contained 25, 50, 100, 200,350, and 500 µL of curcumin niosomal dispersion). Afterward, the mixtures were shaken and placed in a dark area for half an hour at 37 °C. Later on, the levels of DPPH in the products were determined at 425 nm [40]. The following equation was used for the detection of antioxidant activity.

$$Antioxidant\;potential=\frac{\;{Absorbance}_{Blank}-{Absorbance}_{Sample}}{{Absorbance}_{Blank}}\times 100$$

##### Cytotoxicity Studies

Cell viability experiments were employed to assess the biological compatibility of curcumin niosomes. Twenty-four hours before therapy, plates containing 96 wells with full media for proliferation were put in with approximately 103 adult ovarian carcinoma A2780 cell types. Curcumin niosomes were subjected to incubation with the cells, and their responses contrasted those of cells that received PBS and cells treated with 1% (*v*/*v*) Triton X-100 that were used in PBS, representing the two types of controls, accordingly. Following that, cells underwent cultivation for 2 days at a temperature of 37 °C in an environment that was humid and contained 5% CO_2_. Every well received a dose of the dye methylene blue in a 0.01 M borate buffer (pH 8.5). Excessive stain was eliminated after 30 min by rinsing thrice using a 0.01 M borate buffer (pH 8.5). Then, 100 l of 1:1 (*v*/*v*) ethanol and 0.1 M HCL were poured into each well to flush out the dye. The absorption was calculated at 650 nm with an ELISA device after the plates seemed softly agitated [41].

##### Statistical Analysis

Graph Pad Prism was used for statistical analyses and curve fitting. The three separate trials’ observations were combined and given as the mean ± standard deviation. An analysis of variance (ANOVA) in considered one and two dimensions was performed to calculate the statistical levels (*p*-values of 0.05) of category differences. Utilizing Design-Expert program version 13, a Box–Behnken design (BBD) was carried out.

## 4. Conclusions

Curcusomes were synthesized through thin-film hydration for the administration of poorly soluble curcumin and further characterized. The in vitro drug release of curcumin from niosomes presented a continuous steady release. The greater solubility of curcumin in niosomal formulation was responsible for that improved impact. Curcusomes presented more stability; this was attributed to their low PDI. Curcusomes showed higher cell mortality as compared to that with free curcumin. Overall, the results of this investigation indicated that encapsulating curcumin in a niosomal formulation might demonstrate much stronger antioxidant effects and anticancer activity.

## Figures and Tables

**Table 1:** Formulation designed by Box–Behnken design and observed responses for optimization of curcusomes.

Std	Run	Independent Variables			Dependent Variables		
		Chol	Sur: Chol	Cur: Chol	Average Size	PDI	EE
		(μmol)	(*w*/*w*)	(*w*/*w*)	(nm)		(%)
2	1	1	−1	0	356.9 ± 3.19	0.496 ± 0.006	83.6 ± 4.7
12	2	0	1	1	168.4 ± 1.56	0.150 ± 0.002	85.4 ± 3.8
4	3	1	1	0	276.3 ± 1.32	0.228 ± 0.007	86.9 ± 6.5
8	4	1	0	1	318.5 ± 4.25	0.362 ± 0.003	89.4 ± 2.4
15	5	0	0	0	180.9 ± 1.36	0.307 ± 0.006	79.7 ± 3.0
14	6	0	0	0	181.6 ± 1.02	0.318 ± 0.005	78.9 ± 2.9
10	7	0	1	−1	163.7 ± 0.95	0.174 ± 0.002	81.4 ± 4.1
11	8	0	−1	1	215.6 ± 3.11	0.407 ± 0.009	75.9 ± 1.8
13	9	0	0	0	180.5 ± 1.32	0.311 ± 0.004	79.2 ± 4.3
5	10	−1	0	−1	153.9 ± 0.74	0.192 ± 0.006	60.7 ± 5.2
3	11	−1	1	0	142.7 ± 0.92	0.144 ± 0.006	67.2 ± 2.6
9	12	0	−1	−1	163.8 ± 1.15	0.413 ± 0.008	72.1 ± 3.3
7	13	−1	0	1	163.1 ± 1.47	0.217 ± 0.005	64.1 ± 2.8
6	14	1	0	−1	302.7 ± 2.96	0.334 ± 0.003	80.5 ± 2.3
1	15	−1	−1	0	176.2 ± 1.12	0.285 ± 0.004	57.9 ± 1.5

Chol—cholestrol; Sur—surfactant; Cur—curcumin; PDI—polydispersity index; EE—entrapment efficiency. All values represent the mean ± SD; *n* = 3.

**Table 2:** Results of regression analysis for responses Y_1_ (average size), Y_2_ (PDI), and Y_3_ (EE).

Response	Suggested Model	R^2^	Adjusted R^2^	Predicted R^2^	SD	*p*-Value	
Y_1_	Quadratic	0.9968	0.9912	0.9497	0.006	<0.0001	Significant
Y_2_	Quadratic	0.9958	0.9883	0.9491	0.011	<0.0001	Significant
Y_3_	Quadratic	0.9942	0.9896	0.9719	0.009	<0.0001	Significant

**Table 3:** Optimized formulation conditions and responses obtained from RSM data.

Independent Variables	Units	Optimal Values	
A: Chol	μmol	325	
B: Sur–Chol	*w*/*w*	2	
C: Cur–Chol	*w*/*w*	0.5	
**Responses**	**Predicted Value**	**Experimental Value**	**Residual**
Size	165.2	169.4	3.2
PDI	0.192	0.189	−0.003
EE	84.51	84.50	−0.01

**Table 4:** Different levels for independent variables in the RSM (Box–Behnken design) for curcusomes’ optimization.

Independent Variables	Level		
	Low (−1)	Medium (0)	High (+1)
Chol (μmol)	250	325	400
Sur: Chol (*w*/*w*)	0.5	1.25	2
Cur: Chol (*w*/*w*)	0.25	0.375	0.5
**Dependent Variables**	**Goal**		
Average size (nm)	Minimum		
PDI	Minimum		
EE (%)	Maximum		

## Data Availability

All the study data of this manuscript is contained in the manuscript and Supplementary Materials.

## Supplementary Materials

The following supporting information can be downloaded at: https://www.mdpi.com/article/10.3390/ph16101406/s1, Figure S1: (A) Size distribution; (B) Surface charge of curcusomes.
