# Supplementary material for: Formulation and Characterization of Curcumin Niosomes: Antioxidant and Cytotoxicity Studies
Source: Pharmaceuticals (Basel). 2023 Oct 3;16(10):1406. doi: 10.3390/ph16101406 (PMC10610541; doi:10.3390/ph16101406)
Supplement: Supplementary file 1 [file pharmaceuticals-16-01406-s001.zip › pharmaceuticals-2583563-supplementary.pdf]

Formulation and Characterization of Curcumin Niosomes: Antioxidant and Cytotoxicity Studies

Supplementary Figure-S1

A. Size

Results

			Size (d.nm):	% Intensity:	St Dev (d.n...
Z-Average (d.nm):	168.4	Peak 1:	146.0	100.0	55.08
PdI:	0.150	Peak 2:	0.000	0.0	0.000
Intercept:	0.609	Peak 3:	0.000	0.0	0.000
Result quality :	Good				

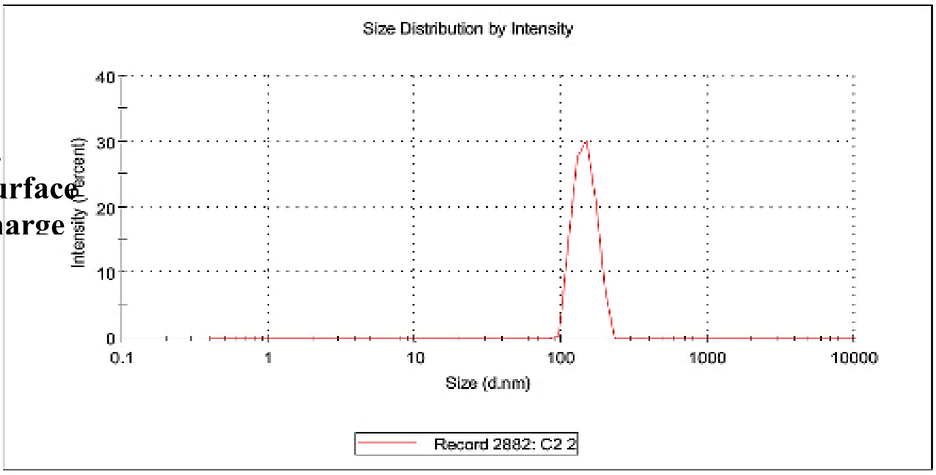

B. Surface charge

Results

			Mean (mV)	Area (%)	St Dev (mV)
Zeta Potential (mV):	-50.6	Peak 1:	-50.6	100.0	7.81
Zeta Deviation (mV):	7.81	Peak 2:	0.00	0.0	0.00
Conductivity (mS/cm):	0.740	Peak 3:	0.00	0.0	0.00
Result quality :	Good				

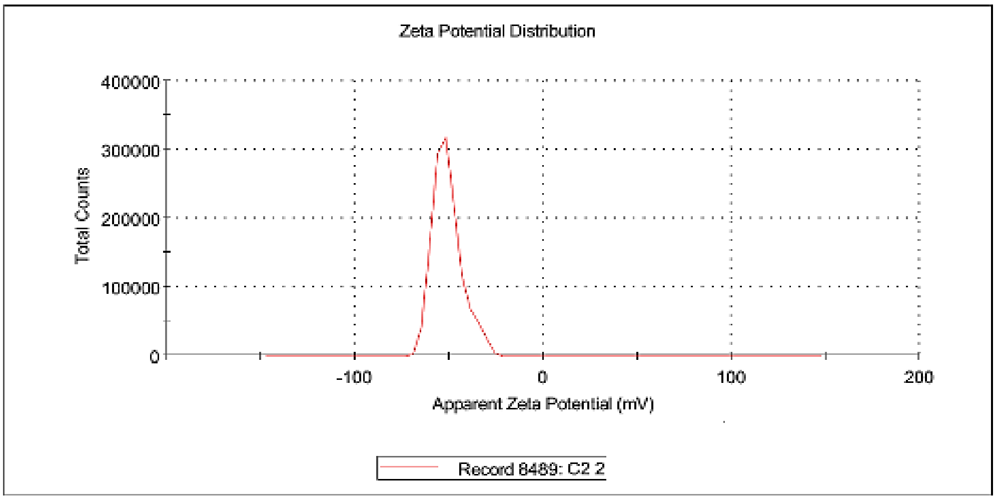

Figure S1. (A) Size distribution; (B) Surface charge of curcusomes
